# Growing cocoa in semi-arid climate and the rhythmicity of stem growth and leaf flushing determined by dendrometers

**DOI:** 10.1016/j.heliyon.2024.e32266

**Published:** 2024-05-31

**Authors:** Thainná Waldburger, Thomas Anken, Achim Walter, Hassan-Roland Nasser, Philippe Monney, Marianne Cockburn

**Affiliations:** a Agroscope, Competitiveness and System Evaluation, Tänikon1, 8356, Ettenhausen, Switzerland; b Institute of Agricultural Sciences, ETH Zürich, Universitätstrasse 2, 8092, Zurich, Switzerland; c Agroscope, Fruit Production in the Alpine Region, Route des Eterpys 18, 1964, Conthey, Switzerland; d Agroscope, Animals and Animal Products, Equines, Les Longs-Prés, 1580, Avenches, Switzerland

**Keywords:** Yield parameters, Semi-arid climate, Cocoa, Dendrometer

## Abstract

This study investigated the performance of cocoa trees within an irrigated cocoa plantation situated in the semi-arid region of Bahia, Brazil. Two treatments were compared: “full sun,” where cocoa trees were not shaded, and “shade,” where trees were covered with a shading net absorbing 30 % of the radiation. The number of leaves and the leaf area index (LAI) were assessed using destructive method on 8 trees. In addition, new flushing of leaves, categorized into four flushing stages, were assessed visually on a weekly basis during two years. The variation of the stem diameter was measured using dendrometer sensors (n = 12 trees). Yield parameters like dry bean yield and number of fruits (healthy and aborted) were assessed on 40 trees per treatment. Both treatments, performed well in the semi-arid region. Generative parameters, such as dry bean yield (±2,000 kg/ha), fruit healthy and abortion rate per plot, were unaffected by full sun and shade treatments. The treatments showed high fruit abortion rates of (±60 %), showing that there's still much room for yield optimization. Additionally, stem diameter of the trees showed a significant reduction of the stem growth (daily increase of stem diameter) and maximum daily shrinkage (daily variation of stem diameter) during the flushing of new leaves. This implies that the emergence of new leaves significantly influences stem growth, consequently affecting the fruits which are growing on the stem. This assumption was corroborated by the significantly increased fruit abortion rate during the flushing of new leaves (stages 1 & 2). These findings highlight the potential of dendrometers to quantify this effect what can be used in future to optimize management practices. By doing so, more effective strategies can be developed to enhance cocoa yield and overall productivity in semi-arid regions.

## 1 Introduction

The production of cocoa beans serves as the primary income for many Brazilian farmers, making cocoa a vital commodity in Brazil, with more than 580,000 hectares under cultivation [1]. Traditionally, cocoa is grown in subtropical regions under shaded conditions [2,3]. Shaded cultivation was deemed a low-cost agroforestry system. However, shade cover leads to lower yields due to the increased competition for light with the shading plants [4]. In addition, the extra layers of leaves that shade the cocoa trees are promoting a humid microclimate, as evidenced in other crops [5]. This favours the development of fungal diseases like *Moniliophthora perniciosa*, responsible for the "witches' broom” disease, which was mainly the cause of the production decline of cocoa in Brazil in the years 1990 [6]. As consequence, farmers started to remove the shading cover to reduce disease pressure and improve cocoa tree productivity, following in the footsteps of other cocoa growing countries, such as Ghana [7]. Many were switching to cropping system with less shade or even full sun cocoa monoculture systems without any shading plants [8]. Moreover, a study carried out in Indonesia showed that it was possible to increase productivity with the reduction of shade tree cover from 80 % to 40 % [9]. Encouraged by promising yields in non-shaded subtropical areas, producers started to expand production to non-traditional regions like the Brazilian semi-arid zone [10]. However, there is still a lack of information on how efficient cocoa production is in the semi-arid climate and how cultivation systems need to be adapted to this dry and hot environment [11].

The development of cocoa is known to have alternating stages of leaf flushing (releases of new leaves) and shoot elongation (root and stem growth) [12]. The flushing last 10–15 days on average [13] and occur 3 or 4 times per year (Oct/Nov, Dez/Jan, Mar) being activated by climatic conditions (air humidity) or soil moisture [12]. The flushing are mainly divided into four different stages, starting with bud swelling and beginning of leaf development (stage 1), followed by leaf expansion characterized by very thin leaves (stage 2), then full leaf expansion with a light green colour (stage 3) and finally fully developed leaves with a dark green colour and dormant apical bud (stage 0 – mature leaves) [14]. During the leaf flushing the concentration of soluble carbohydrates and starch in old mature leaves, roots and stems decreased. This is mainly observed during stage 2, which is therefore considered as the key period of carbohydrate competition with growing fruits [12]. The growth of the expanding leaves, which have no active photosynthesis, depend on the carbohydrates produced by mature leaves and are therefore an important sink [15,16]. The dependency decreases once the new leaves have been fully developed (stage 0) and can therefore, produce and export photoassimilates [17]. After the leaves are developed, stem and root development increase again [16]. Furthermore, studies have revealed a decrease in nitrogen content in old mature leaves during this period [15,13]. This decline has been linked to a reduction in the enzyme rubisco, resulting in decreased photosynthetic activity [15]. Lahive et al. [18] emphasized that current knowledge regarding the dynamics of carbon allocation in cocoa primarily focuses on partitioning between above- and below-ground organs. Little attention has been paid to allocation patterns between vegetative and generative organs. The disparity between vegetative and generative growth can prompt the plant to abort fruit as a compensatory measure. It is already established that the plant has a compensatory measure that can be triggered by limited carbohydrate availability and competition among fruits [19]. A higher rate of fruit abortion may also occur when the tree is experiencing intense flushing stages [19].

Dendrometers are sensors measuring continuously the stem-diameter and are widely used for evaluating stem growth and water stress in plants [20]. In addition, dendrometer data provide valuable insights into the tree's response to local climatic conditions [21]. The diurnal stem-size variation is used to characterize plant's reactions to climatical conditions, changing soil water content and rainfall [22,23]. The daily pattern of dendrometer data is delineated by two stages: (i) the contraction phase, occurring between daily maximum at sunrise and daily minimum at sunset; (ii) the expansion phase, spanning from the daily minimum to the following maximum [24,25]. The periodicity of shoot elongation, root growth and fruit load has been associated with variation in stem diameter in avocado trees [26]. Waldburger et al. [27] showed that dendrometers are suitable to characterize the growing cycles of cocoa too. However, the intricate relationship between stem growth, leaf flushing and fruit set remains incompletely understood [27], emphasizing the necessity for a study exploring the rhythmicity of vegetative growth in relation to generative growth in cocoa trees.

This study aimed to assess the influence of phenological factors on the performance and growth cycles of cocoa trees in a semi-arid climate. The research also considered the influence of full sun and shade treatments on cocoa trees, along with the effects of new flushing stages on stem growth and fruit abortion.

## 2 Materials and methods

### 2.1 Site

**Fig. 1:**
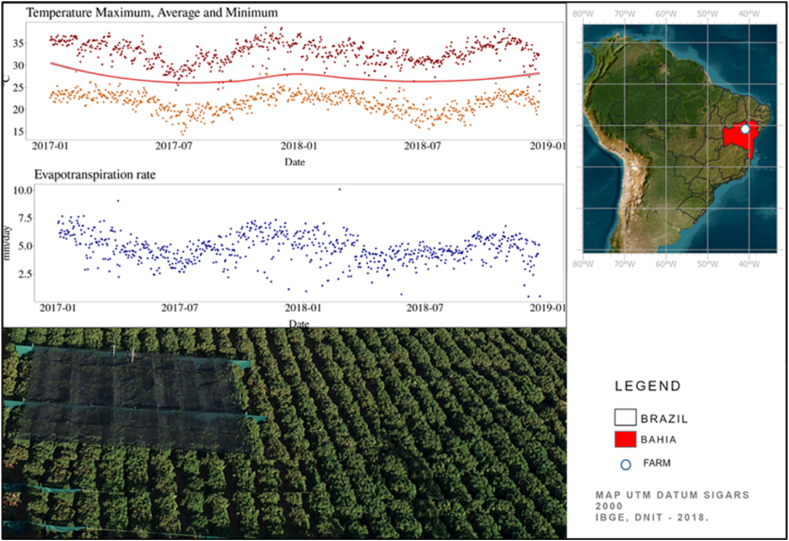
Maximum (point in dark red), average (line in red) and minimum (point in orange) temperatures (top right) and the reference evapotranspiration rate (bottom) on the farm during 2017 and 2018. The plantation area with the plots in the windbreak + shading area (left) and part in full sun (right) of the CCN51 variety. At the top right is a map of Brazil (black line), the location of the state of Bahia (red) and a white circle representing the study farm.

A field trial was conducted from 2017 to 2018 in Juazeiro, in the semi-arid region of Bahia, Brazil (coordinates: −9.122°–40.258° at sea level) (Fig. 1). The climate of the region is characterized by high average annual temperatures (24.8 °C) and low humidity (daily average below 65 %), which results in high evapotranspiration rates (Fig. 1), classified as “hot semi-arid” [28]. The average annual rainfall is 422 mm.

Soil texture was characterized by approximately 15 % clay, 15 % silt, 70 % sand and 1.5 % organic matter, leading to a classification as sandy loam (0–25 cm of depth). The layer below 30 cm depth was compact and exhibited low permeability. Irrigation was performed by the farm, aiming to keep the soil matric potential between - 30 KPa and - 60 KPa, and - 20 KPa to - 40 KPa, respectively for soil (20 cm) and sub-soil (40 cm). The trees were irrigated with two dripper lines per tree line (1.0 L h^−1^ flow rate and a distance of 50 cm between the drippers). The trees developed roots to a depth of 25 cm mainly concentrated below the dripper lines. Owing to the shallow soil, the irrigation was split to two to three pulses per day.

The farm applied drip irrigation fertigation. No fungicide treatments were applied.

### 2.2 Experimental setup

In the current study, 80 cocoa trees of the variety Colección Castro Naranjal 51 (CCN 51) planted in the year 2014, were evaluated. The plant density was 1,250 trees per hectare with a row spacing of 4 m and a distance between the trees of 2 m.

#### 2.2.1 Full sun and a shade treatment

Trees were planted in the shade of bananas, which were removed in year 2016 ending in plantation without shading called “full sun”. In the shade treatment, trees were covered by a shading net (sombrite, ABNT NBR 15560-3, equipesca, Brazil) that absorbed 30 % of solar radiation and provided protection from wind. Each treatment consisted of four individual plots placed in one strip. The plots of the treatments were arranged pairwise (blocks) allowing direct comparisons. Each single plot consisted of 10 individual trees (80 m^2^) and two trees in the end of the row to avoid border effects. Individual trees serving as pseudo-replicates were averaged for each plot for the calculation of yield, healthy as aborted fruits and leave flushing. Leaf area index (LAI) was determined by cutting 4 trees from each treatment. These trees were completely defoliated, and the total number of leaves and the length of every 10th leaf was recorded. To determine the relation between the length and the leaf surface, the length and area of 35 single leaves from each treatment was determined. The 70 leaves were photographed from a constant distance. The number of pixels were counted per leaf using computer vision. By determining the size of a single pixel, the leaf area was calculated using the histogram tool of the Adobe Photoshop 9.0 software (Adobe, San Jose, CA). Based on the regression derived from 70 single leaf surfaces and their length (Equation (1)), the total leaf area of each tree was calculated. Finally, the LAI was determined by dividing the leaf area per tree by the ground surface occupied by each tree (8 m^2^).

(1) $$y=0.25x^{2}\;-\;1.07x+9\;\left(r^{2}=0.97\right)$$

y = Leaf surface (cm^2^)

x = length (cm)

#### 2.2.2 Yield parameters

**Fig. 2:**
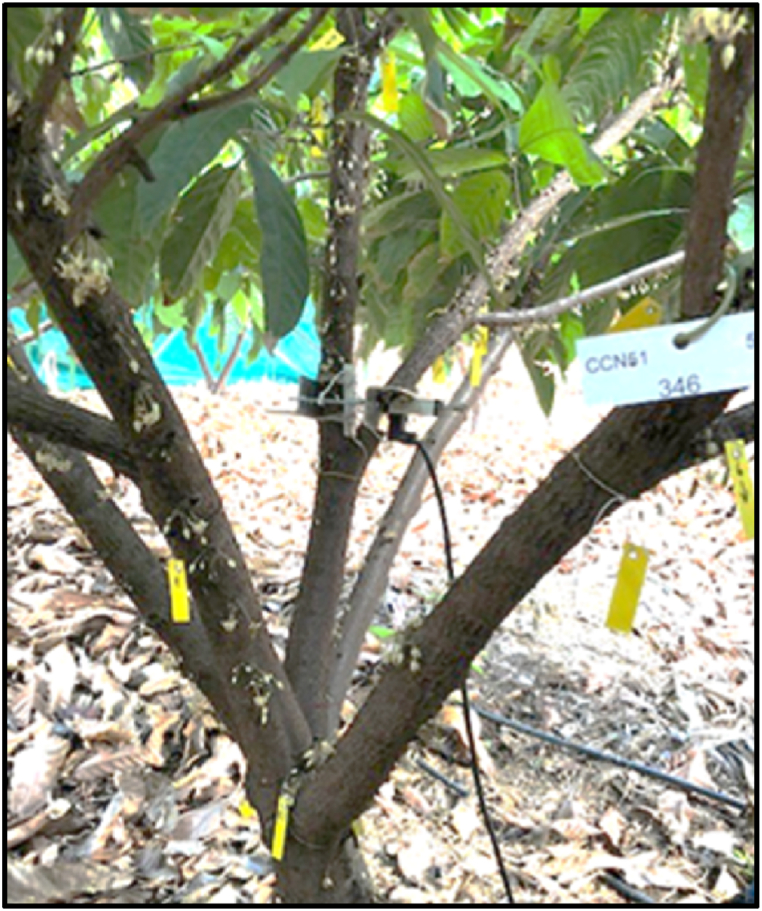
Cocoa tree equipped with a dendrometer sensor and labels dividing the branches for weekly fruit counting.(2)drybeanyield(kg)=freshfruitweight(kg)*0.14

The yield parameters were recorded for 10 trees per plot, resulting in a total of 40 trees per treatment. All healthy and aborted (dry) fruits were counted manually once per month for all 40 trees per treatment. For trees equipped with dendrometers (12 trees in total) the counts were performed every week (Fig. 2). Only ripe fruits were harvested and weighed for each plot at the corresponding date. Ripe fruits were harvested from October 2017 to September 2018. The harvest days were: 2017 = 03/10; 15/11; 12/12, 2018 = 30/01; 15/02; 07/03; 12/04; 29/05; 29/06; 31/07; 28/08; 27/09. The dry bean yield, measured in kilograms, was calculated from the fresh fruit weight using Equation (2). This equation was established on-site by gaining dried kernels out of 20 fresh fruits. No differentiation by treatment was made at this step. The water content of the kernels was set to 6 %.

#### 2.2.3 Flushing stages

**Fig. 3:**
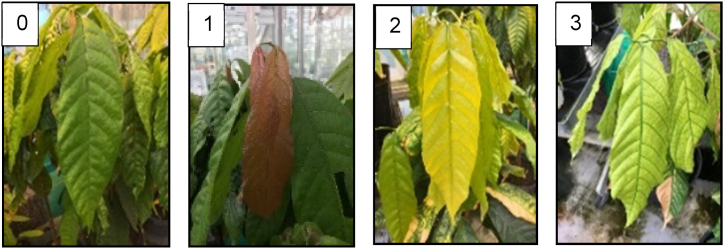
Visual scoring performed on the field for generative development. Flushing stages were divided in four stages (stage 0: no flushing; stage 1: brown leaves; stage 2: yellow leaves; stage 3: light green leaves).

To monitor the vegetative development of the trees, flush stages were visually scored weekly on 12 trees (with dendrometer sensors) and monthly on the other trees in the experiment (without dendrometer sensors). As trees typically have leaves at different stages of development, the dominant stage was considered for evaluation. Leaf flushing stages were categorized into four development stages (Fig. 3). In stage 0, no flushing occurs, stage 1, the leaves are brown. In stage 2, the leaves turn yellow, and in stage 3, they turn green.

#### 2.2.4 Dendrometers

**Fig. 4:**
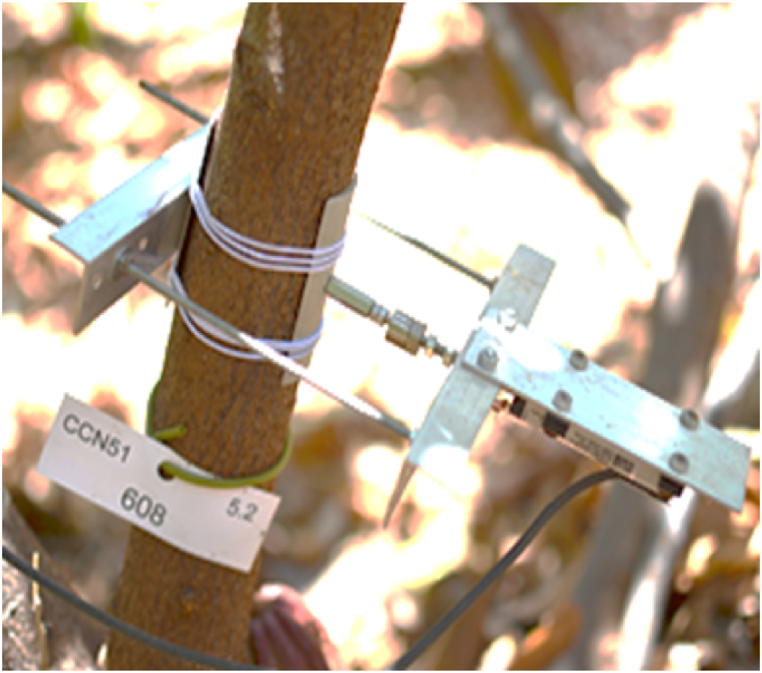
Dendrometer installed on a cocoa tree in Juazeiro, Brazil.

Dendrometer sensors were installed on the stem of the cocoa tree to measure stem diameter, thus providing insights into plant growth dynamics. Stem diameter measurements were conducted from January 2017 to August 2018 for 12 trees belonging to the 80 trees (6 tress in full sun + 6 trees in shades plots) in the experiment. These sensors, as depicted in Fig. 4, were constructed by Agroscope. They consist of a linear potentiometer (Type Megatron, MSLPT 25 mm, Andig, Allinges, France) fixed on an aluminum frame, enabling the “in” and “out” movement of the potentiometer. The sensor achieved a resolution of ±6.1 μm. A rubber band secured the fixation, and dendrometers were affixed to the bark of the trees, oriented facing south to avoid direct solar radiation. Stem-diameter variations were measured every 15 min as a voltage signal and stored by an Agriscope data logger (version 2.327, Agriscope, Mauguio, France). Data transmission occurred via medium wave radio frequency (916 MHz), with a modem transferring the data to an online platform (www.agriscope.fr).

### 2.3 Data processing

The successive contraction and expansion stages of the cocoa stem were analyzed over a 24-h cycle. The data were processed in R version 4.0.1/2 [29] using the dendrometeR package [30] to format the dendrometer data and fill the missing data by using the “fill_gap” and “is.dendro” functions and in addition extract features such as maximum, minimum, and amplitude.

One common issue encountered was the presence of “noise” in the transmitted data curve, often caused by sensor movement on the tree. Curves displaying inconsistencies were optimized by applying filters such as median or mean to smooth them [31].

**Fig. 5:**
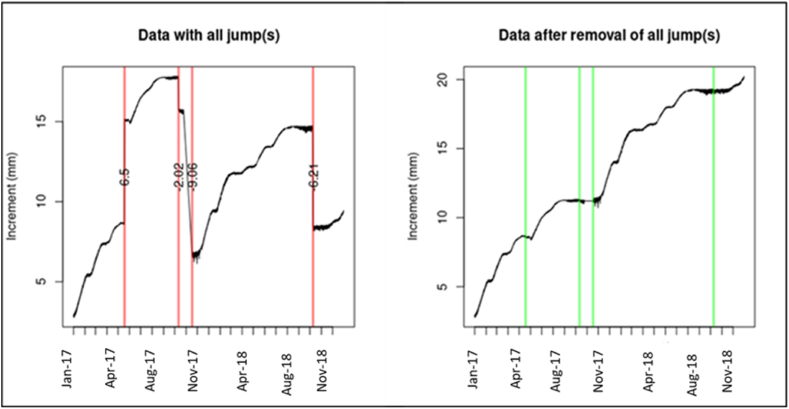
Dendrometer curves of one tree before and after removing jumps.

Additionally, some signal frequency oscillations were observed (Fig. 5), which were corrected by removing shifts in the transmitted signals during short periods using the “jump.locator” function (“dendRoAnalyst” [32]). Each signal was evaluated individually to adapt the baseline-shift value accordingly. The “network.interpolation” or “spline.interpolation” function (“dendRoAnalyst” [32]) was utilized to correct data by extrapolation, addressing accidental displacements of the sensors.

All information about the stages of the daily variation, duration, start and end, as well as maximum and minimum values were obtained with the “daily.data*”* function (“dendRoAnalyst” [32]). Once the parameters had been obtained, daily growth and maximum daily shrinkage were calculated (Equation (3) and Equation (4)).

(3) $$\text{Daily}\;\text{Growth}\;\left(\text{DG}\right)=\left(\;{\text{Maximum}\;\text{daily}\;\text{value}}_{\left(\text{Day}\;-\;1\right)}\;-\;{\text{Maximum}\;\text{daily}\;\text{value}}_{\left(\text{Day}\right)}\right)$$

(4) $$\text{Maximum}\;\text{Daily}\;\text{shrinkage}\;(\text{MDS})=\left(\;{\text{Maximum}\;\text{daily}\;\text{value}}_{\left(\text{Day}\;\right)}\;-\;{\text{Minimum}\;\text{daily}\;\text{value}}_{\left(\text{Day}\right)}\right)$$

#### 2.3.1 Statistics

Descriptive statistics were performed to report production parameters from the treatments full sun and shade by means of *t*-test to compare the means of two groups. The same test was made by the fruit abortion rate in relation to flushes. A linear mixed-effects model was obtained for flush stages and daily stem diameter.

##### 2.3.1.1 Model processing

A linear mixed-effects model (“lmer” method; [33]) from the lme4 package, was performed to analyze the relation of the daily stem-size variation and flushing stages of the 12 trees. The fixed and random effects were chosen and implemented in a model as a function of daily growth and maximum daily shrinkage. After both models were processed, the residuals were checked graphically for normal distribution and homogeneity of variance (supplementary data). After analyzing the residuals, the maximum daily shrinkage data was transformed using the square-root function. The package stargazer [34] was also used to computing p-values and confidence intervals.

Daily Growth Model

Dailygrowth(continuous)∼Flushing(factorwith4levels)+(1|Week/Treelabel)+(1|Treatment)

Maximum daily shrinkage Model

sqrt(Maximumdailyshrinkage(continuous))∼Flushing(factorwith4levels)+

(1|Week/Treelabel)+(1|Treatment)

##### *2.3.1.2* Model selection

The criterion used to select the best model was based on the Bayesian information criterion (BIC). This methodology allows obtaining measures of model performance by assuming that the model errors are independent and normally distributed [35]. The selection via BIC presents an alternative to the p-value test, since it allows a statement to be made about the alternative hypothesis, rather than just the null hypothesis [35]. The “dredge” function from the MuMIn package was used to find the best model based on the smallest BIC value with the largest model weight (w) [36]. Hereby, the model weight gives an indication of the probability that the specific model is optimal in a set of all considered models, where the weight of all calculated models adds up to 1. If the difference in BIC or Akaike information criterion (AIC) (delta) between the best and the second-best model is below 2, the simpler model should be chosen. The AIC was additionally used to cross-check the similar probability between the chosen models [37, 38].

##### 2.3.1.3 Treatments divergence

A one-sample *t*-test was computed to determine the statistical differences between the generative growth (yield parameters) and vegetative growth (LAI and number of leaves) per treatment (R packages: stats, rstatix).

## 3 Results

The performance of cocoa trees was observed considering the difference between the two treatments full sun and shade. The relationship between flushing stages and stem diameter was observed in all 12 trees (full sun and shaded).

### 3.1 Yield parameters and dry bean yield

**Fig. 6:**
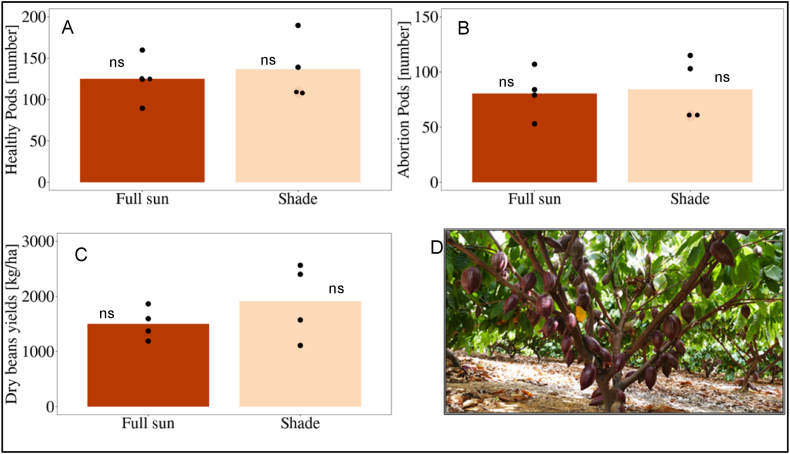
A) Average number of healthy pods from 80 trees in the two treatments for one year (Oct/2017 until Sep/2018). B) Average number of aborted pods from 80 trees in the two treatments for one year (Oct/2017 until Sep/2018). C) Average dry bean yield (kg/ha) of 4 plots with 10 trees each from one year (Oct/2017 until Sep/2018) in the two treatments full sun and shade. D) Cocoa tree in the field in Juazeiro, Brazil. ns = not significant (t-tests). The black dots indicate the average of one single plot.

Full sun and shade treatments did not affect any yield parameters. The number of healthy pods in the shade treatment ranged from 109 to 190 per tree with an average of 137 healthy pods, on the other hand, the full sun treatment ranged from 90 to 160 with an average of 130 number of healthy pods per tree. Regarding aborted fruit per tree, both treatment showed in average nearly the same number of aborted fruits which was at a level of about 60 % aborted fruits for both treatments (Fig. 6).

The yields, as the sum of the different harvests of one year (Oct 2017–Sep 2018), were in average higher in the shade (1.9 t/ha year) than in the full sun treatment (1.5 t/ha year) (Fig. 6). Due to the large variation of the single plots (Fig. 6), the difference of 21 % was statistically not significant. The maximum yields in the shade treatment reached a level of 2,565 kg dry beans per hectare (sd = 659 kg dry beans per hectare).

### 3.2 Leaf area per treatment

**Fig. 7:**
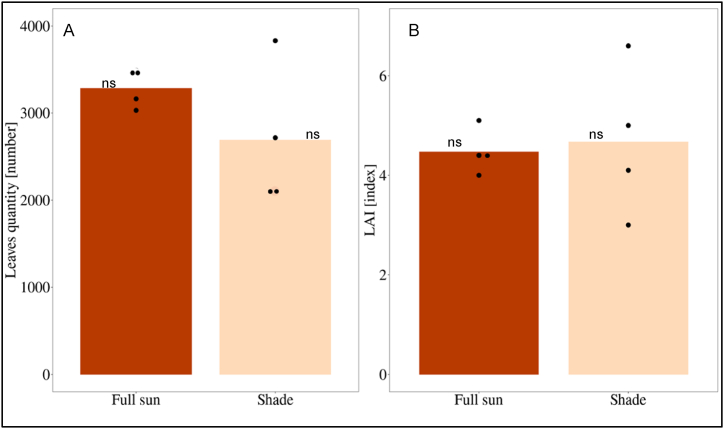
A) Average of total number of leaves per tree per treatment. B) Average of Leaf area index (LAI) per tree per treatment. Average per 4 trees per treatment. The black dots indicate the values of one single tree. * = significant; ns = not significant).

The trees in full sun had an average of 3286 leaves per tree whereas those in the shade treatment had 2694 leaves (Fig. 7 - A). No significant difference between treatments was observed for the number of leaves per tree and LAI (Fig. 7-A and B).

### 3.3 Stem growth and variation determined by dendrometers

**Fig. 8:**
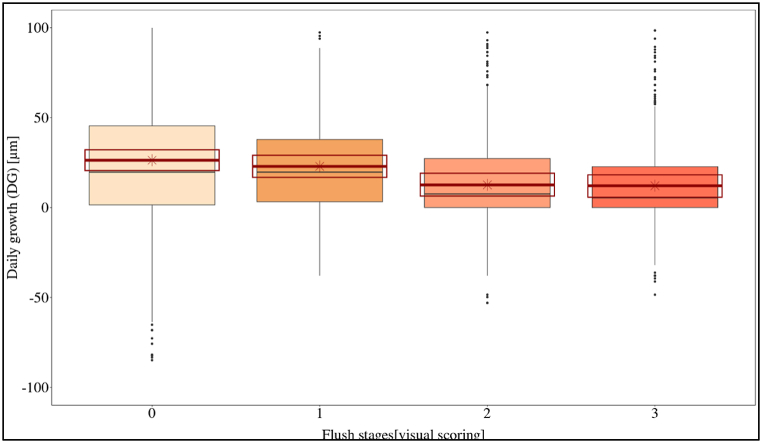
Daily growth (DG) in relation to flush stages 0–3. The boxplot and point (outliers) represent the raw data with the median. The upper end of the box indicates the 75th percentile and the lower indicates the 25th percentile. The red boxplot area represents the model prediction with upper and lower 95 % confidence interval and the median star point.

**Fig. 9:**
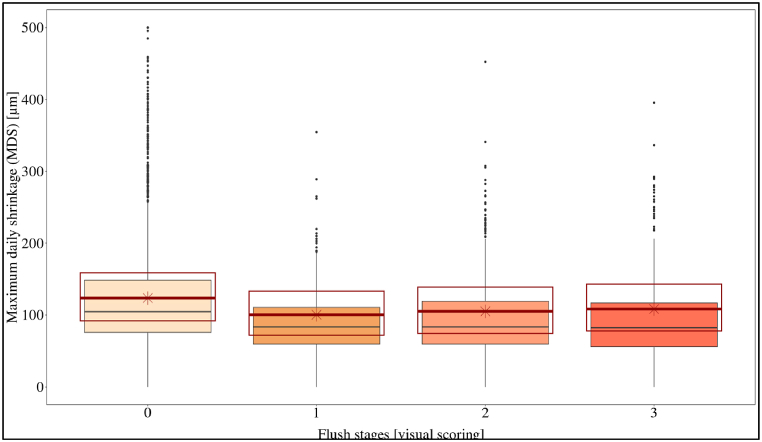
Maximum daily shrinkage (MDS) in relation to flushing. The boxplot and point (outliers) represent the raw data with the median. The upper end of the box indicates the 75th percentile and the lower indicates the 25th percentile. The red boxplot area represents the model prediction with upper and lower 95 % confidence interval and the median star point.

By means of dendrometers, the stem diameter has been measured continuously from January 2017 to August 2018. All trees showed a similar pattern with the start of stem shrinkage at sunrise and start of stem swelling around sunset. Despite showing the same pattern, the dendrometer data showed a high coefficient of variation (CV > 50 %). Daily growth of the 12 trees, defined as the daily growth of the stem diameter, was correlated to the leaf flushing stages (Fig. 8). The results of the daily growth model show that stem growth decreased with increasing flushing stage and mostly took place during non-flushing periods (Table 1; Table 2). On average, the whole flushing stages (stages 1–3) lasted one to two weeks and the non-flushing period lasted three to four weeks. The maximum daily shrinkage (MDS) also decreased with the onset of flushing with a maximum value of 889.61 μm (Fig. 9; Table 1; Table 2).

**Table 1:** Results from the two models performed to evaluate daily growth and maximum daily shrinkage during flushing stages.

Target variable	Fixed effect	Random effect	BIC^a^	AIC^b^	wi^c^
Daily growth	flushing	week/Tree label	39974	39924	1
			40057	40025	0
Maximum Daily Shrinkage	flushing	week/Tree label	19614	19719	1
			19675	19800	0

a Bayesian information criterion.

b Akaike information criterion.

c The model weight (wi) shows the probability that the chosen model is the best in all possibilities evaluated.

**Table 2:** Data estimated based on the model assumption (Daily growth x Flush/Maximum daily shrinkage x Flush).

Flushing stage	Daily growth			Maximum Daily Shrinkage		
	estim	Lo.ci	Up.ci	estim	Lo.ci	Up.ci
0	26.3**	20.6	32.1	123.5**	91.3	160.5
1	22.9*	16.8	29.1	100.0	70.1	132.4
2	12.6**	6.4	19.1	105.0	74.4	139.1
3	12.1**	5.7	18.2	108.5*	78.1	143.1

Signif.: ‘**’ 0.01 ‘*’ 0.05 ‘.’ 0.1 ‘’ 1.

Lo.ci lower 95 % confidence interval.

Up.ci upper 95 % confidence interval.

**Fig. 10:**
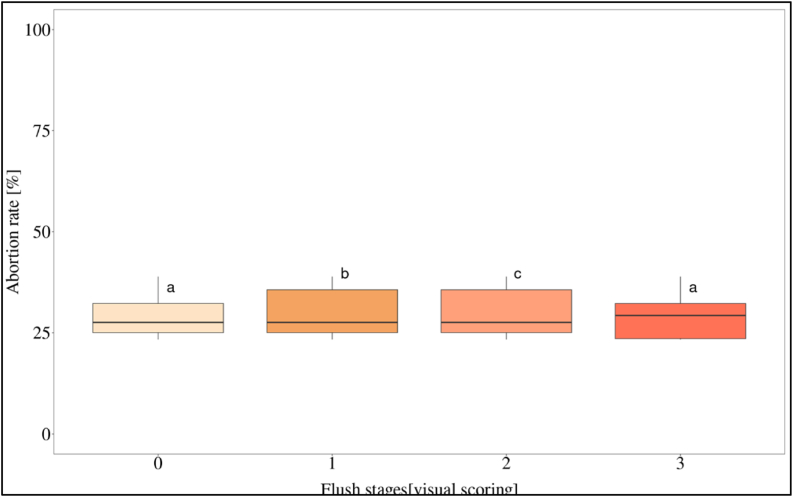
The relation between abortion rates (average per plot) and flushing stages. The boxplot represent the raw data and the median with just the 75th percentile. 100 % corresponds to the total number of aborted fruits of a tree.

In addition, the flushing influenced the abortion rate per plot (Fig. 10). The trees exhibited reduced fruit loss during non-flushing periods and as the leaves reached the final stages of development (stages 0 & 3). The highest number of abortions was found when the leaves were expanding (stages 1 & 2).

## 4 Discussion

In the dry and hot semi-arid regions of Brazil, cocoa trees performed very well. The CCN 51 variety, renowned for its vigor, high productivity, and elevated abortion rate [39] yielded approximately 2,000 kg of dry beans per hectare and year (Fig. 6), a performance comparable to that observed in humid tropical climates [40]. Leite et al. [41], documented yields of 2,260 kg/ha in the same semi-arid region of Brazil, consistent with the findings of our study.

The vegetative growth of cocoa did not exhibit significant differences between treatments shade and full sun in terms of number of leaves per tree, leaf area index (LAI), number of fruits (healthy and aborted) or dry bean yield. The LAI is comparable to the one indicated in full sun plots [42]. The best shaded plots showed yields up to nearly 2,500 kg dry beans/ha (Fig. 6) indicating the potential of the crop grown in semi-arid conditions. Due to the high variability among individual plots, the averages of the two treatments were not statistically significant. This fact is attributed to variable soil conditions, with lower areas of the field prone to waterlogging during periods of heavy rainfall.

Dendrometers provided insights into the daily pattern of stem contraction and expansion. Two models depicted the rhythmicity of the stem diameter in relation to the new flushing of leaves (Fig. 8, Fig. 9). In the first model the trees exhibited a notable decrease in stem growth during flushing (Fig. 8). Daily growth started to decrease when new leaves began to flush (stages 1, 2 and 3) and subsequently increased once the leaves were fully developed and no flushing was occurring (stage 0). The model illustrated that all flush stages significantly affected stem growth (p < 0.05). The reduction in stem growth was induced by the flushing (stages 1 and 2), representing a sink for carbohydrates [12], a demand that gradually diminishes as the leaves mature and start photosynthesis [15,13]. Dendrometers are well capable to characterize these growing stages.

A second model showed that maximum daily shrinkage was also reduced during the flush stages (Fig. 9), indicating a lower evapotranspiration rate and thus a decrease of the photosynthesis [43]. This was unexpected, as the existing leaves remained active, and the upright position of the small new leaves (stages 1) did not result in a strong shading of the active leaves. A plausible explanation has been provided by Sleigh et al. [16]: mature old leaves exhibit a lower photosynthetic capacity due to lower concentrations of the soluble and starch carbohydrate in the leaves during the flushing of new leaves. Consequently, the sink for soluble compounds (N,P,K) and starch carbohydrate of the new leaves may induce the reduction of the photosynthesis of the old leaves. It can be presumed that this reduced availability of carbohydrates correlates with decreased stem growth, evidenced by the dendrometer sensors.

Dendrometer data revealed considerable variability among individual trees. Factors such as the method of fixation and positioning on the tree, as well as cable connections, may have contributed to reduced data quality. Enhancements in dendrometer fixation methods could help mitigate this variability. Despite these challenges, dendrometers adeptly capture the relationship between phenological stages and cocoa growth rhythm, serving as invaluable monitoring tools for advancing cocoa plantation management practices.

The abortion of new fruits reached a high level of 60 %, showing the potential loss of yield. Such high abortion rates are not exceptional and also cited in humid regions [44]. Fig. 6 shows a very high variability of the abortion, which has already been proven in other study [45]. A deeper understanding of the factors contributing to the variability in abortion rates among trees could enhance management strategies.

The abortion of new fruits increased significantly during the flushing of new leaves at the stages 1 & 2 (Fig. 10) what is in accordance with a large study of CEPLAC [46] who observed a coincidence of abortion and flushing. Fruit abortion is described as a plant protection mechanism against nutritional deficit arising from internal competition for carbohydrates among young fruits and fruits of different ages [19]. Since fruits develop on the stem, it appears that fruit abortion is influenced by the emergence of new flushes, as indicated by a concurrent reduction in stem growth. This implies that vegetative development in cocoa representing a significant sink, is potentially leading to a decrease in the plant's generative capacity [47]. The question arises as to how this effect can be mitigated.

Decreasing nitrogen fertilization during the periods without flushing may mitigate vegetative growth, thus reducing competition for carbohydrates. On the other hand, an increased fertilization during the flushing to reduce the decrease of the nutrients (N, P, K) in the existing leaves might reduce the abortion of fruits during this critical period of fruit setting. How to effectively manage this behavior of cocoa trees remains an area for further investigation. Nonetheless, cocoa has demonstrated promising results in the semi-arid region, and data collected through dendrometer sensors have facilitated the real time measurement of phenological characteristics.

## 5 Conclusions

Cocoa trees performed remarkably well in the semi-arid region of Brazil, exhibiting higher yields (2000 kg dry beans/ha) than those typically observed in traditional regions. This shows the high flexibility of the plant normally grown in humid climates. Similarly, to the traditional region cocoa has shown alternating rhythms of flushing and stem growth. Dendrometers are well suited to show the growth rhythmicity of cocoa trees. Their data revealed a decrease in stem development during the flushing of new leaves, resulting in a significant increase in the abortion rate. Furthermore, this reduction in stem growth and maximum daily shrinkage expresses a decrease in the evapotranspiration rate. These findings are in line with the literature describing the competition for carbohydrates and nutritional elements (N, P, K in leaves) between new leave flushing and stem/root growth, with priority given to new leaves. As fruits grow directly on the stem and compete for carbohydrates, a logical next step could involve adjusting fertilization strategies to better align with growth stages. Increasing fertilization during key periods of fruit abortion, complemented by strategic pruning, may enhance productivity. The integration of dendrometer sensors offered real-time insights into the primary growth cycles of the trees. Looking ahead, leveraging these sensors could enable the optimization of crop management across various growth stages, effectively minimizing the risk of fruit loss—a crucial factor contributing to yield reduction.

## CRediT authorship contribution statement

**Thainná Waldburger:** Writing – review & editing, Writing – original draft, Validation, Formal analysis, Data curation, Conceptualization. **Thomas Anken:** Writing – review & editing, Writing – original draft, Visualization, Validation, Supervision, Methodology, Formal analysis, Data curation, Conceptualization. **Achim Walter:** Writing – review & editing, Writing – original draft, Visualization, Supervision. **Hassan-Roland Nasser:** Visualization, Validation, Methodology, Investigation, Formal analysis, Data curation. **Philippe Monney:** Writing – original draft, Validation, Supervision, Formal analysis, Conceptualization. **Marianne Cockburn:** Writing – review & editing, Writing – original draft, Visualization, Validation, Supervision, Methodology, Conceptualization.

## Declaration of competing interest

The authors declare the following financial interests/personal relationships which may be considered as potential competing interests:

Anken, Thomas reports financial support was provided by Innosuisse.
