# Supplementary material for: Growing cocoa in semi-arid climate and the rhythmicity of stem growth and leaf flushing determined by dendrometers
Source: Heliyon. 2024 May 31;10(11):e32266. doi: 10.1016/j.heliyon.2024.e32266 (PMC11214439; doi:10.1016/j.heliyon.2024.e32266)
Supplement: Multimedia component 1 [file mmc1.docx]

# **Supplementary data**

Additional residual analysis in R – with plot () function on the two static models presented.

Statistical results of the Daily growth model (Figure 11)


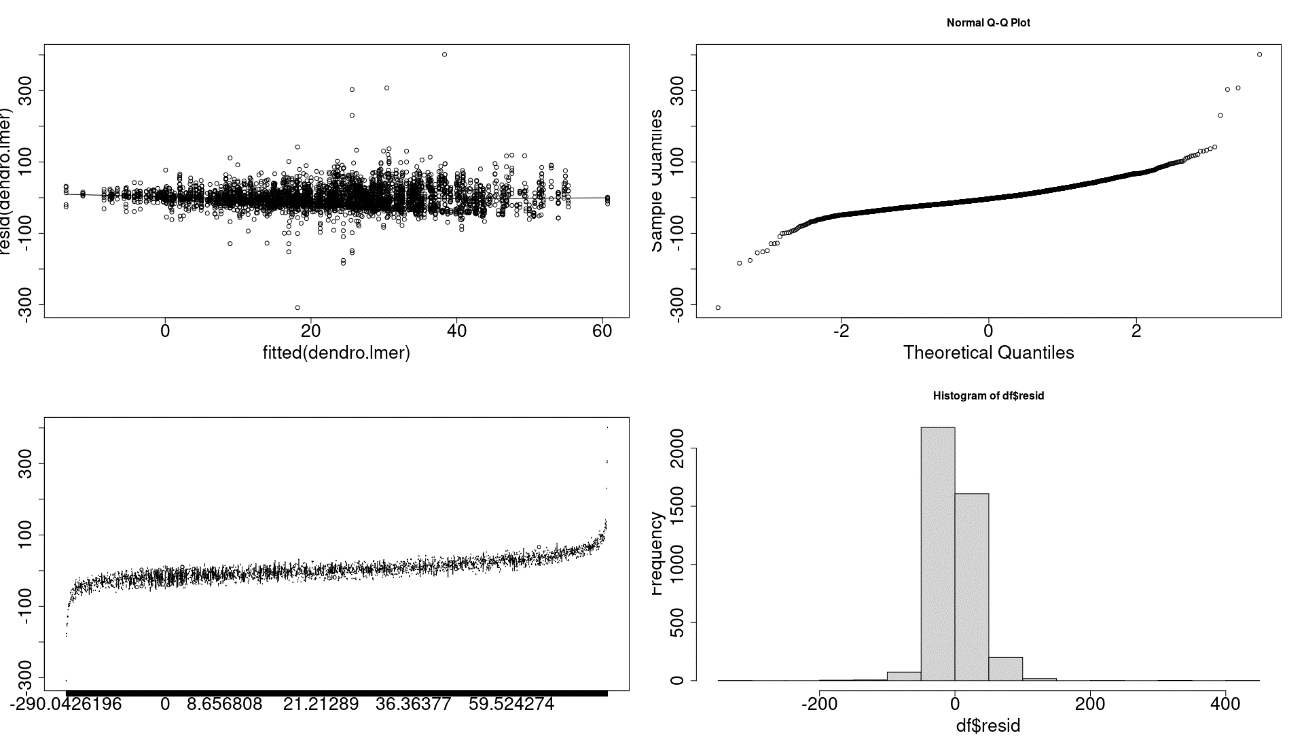


Figure 11 Data on: residuals vs fitted (pearson), Normal Q–Q (normal distribution), boxplot residuals, and histograms.

Statistical results of the Maximum daily shrinkage model (Figure 12)


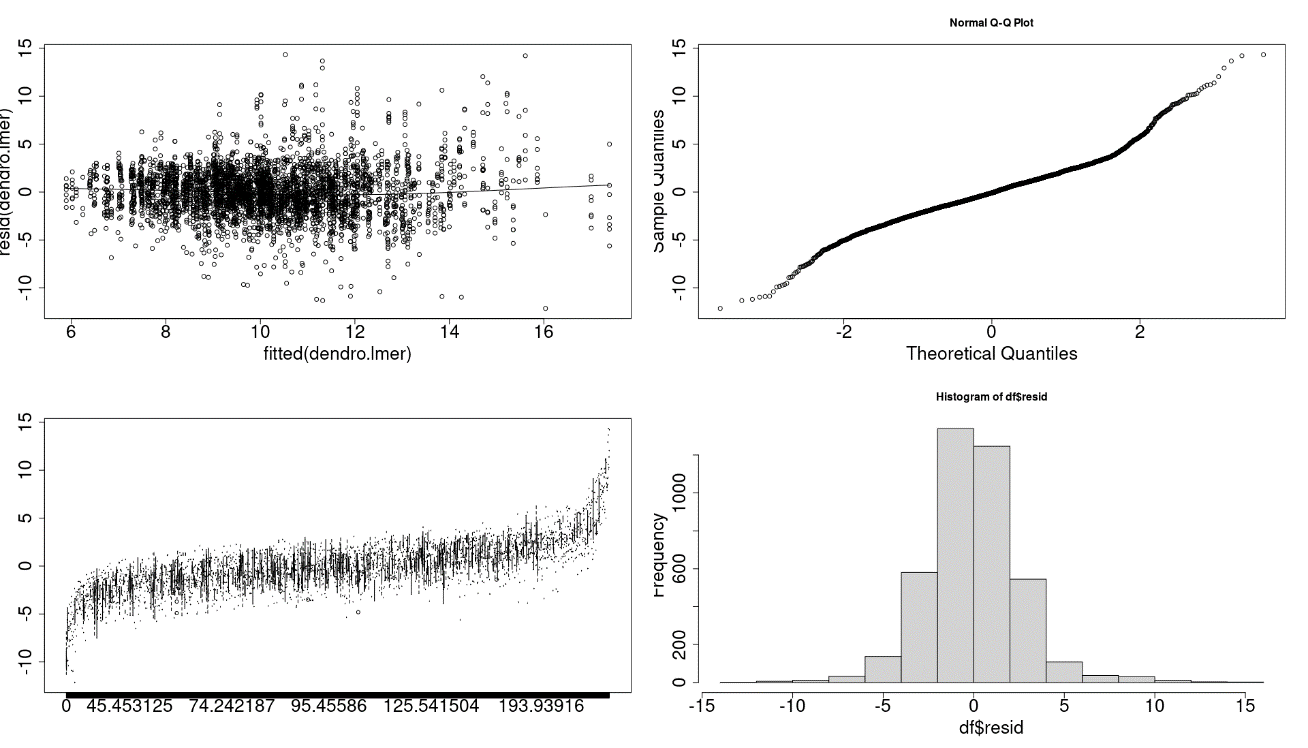


Figure 12 Data on: residuals vs fitted (pearson), Normal Q–Q (normal distribution), boxplot residuals, and histograms.
